# Use of entropy in the analysis of nominal traits in sheep

**DOI:** 10.1007/s13353-012-0123-z

**Published:** 2012-11-23

**Authors:** Anita Dobek, Ryszard Steppa, Krzysztof Moliński, Piotr Ślósarz

**Affiliations:** 1 Department of Mathematical and Statistical Methods, Poznań University of Life Sciences, Wojska Polskiego 28, 60-637 Poznań, Poland; 2 Department of Small Mammal Breeding and Animal Origin Materials, Poznań University of Life Sciences, Złotniki, Słoneczna 1, 62-002 Suchy Las, Poland

**Keywords:** Entropy, Genetic markers, Mutual information, Prolificacy, Sheep

## Abstract

In the analysis of dependencies between nominal traits entropy and its function, mutual information seems to be a proper descriptive statistic. This is shown by characterizing the relationships between the prolificacy of dams and selected genetic attributes: the genotype of transferrin, the genotype of hemoglobin, and the type of birth, as well as the environmental attribute, i.e., year of birth. The entropy method may improve the exactitude of investigations concerning the influence of different factors on production trait. The index of relative uniformity, introduced in this study, proved to be an adequate tool for the determination of similarity in the examined flocks. The application of mutual information in the determination of values of the dependence measures in the analyzed experiment was justified.

## Introduction

Analysis of dependencies between traits is commonly applied in multitrait experiments. The character of observed traits determines the selection of statistical methods used in this analysis. Analyzed traits may be continuous or discrete. One of the categories of discrete traits is nominal traits. These include, e.g., all binary traits, genotype for a specific gene, blood group, etc. In the literature, we may find several methods for determining the values of correlation coefficients for such variables. As a rule, these methods use values of χ^2^ statistics from contingency tables. These include the following coefficients: Tschuprow’s T, Pearson’s C, and Cramer’s V (Sheskin 2004; Liebetrau 1983). The value of χ^2^ statistics is a good measure of the dependence of variables, when no empty cells are found. It is known that the standard χ^2^ test may be applied when the number of classes of less than 5 in size does not constitute 30 % of the total number of classes and, additionally, there are no empty cells (Lancaster 1969). Otherwise, the approach is not adequate, due to the approximations consisting of the substitution of zeroes in the empty cells, with their expected values always being different from zero. However, the small number of observations in subclasses is a very common problem, especially when the number of variables is increasing. In such a situation, a method of entropy constitutes a good tool, as it is based on information being a function of relative frequencies. In the analysis of entropy, the dependence of traits is defined by mutual information (Jakulin and Bratko 2004). Simulation studies provided by Dobek and Moliński (2011) show the correctness of this approach. The entropy-based method is more powerful than conventional methods and can be useful in the detection of epistasis for rare genes (Moore et al. 2006; Kang et al. 2008; Ruiz-Marín et al. 2010). This study presents three indexes of trait dependence as a function of mutual information. Additionally, the index of relative uniformity, being an extension of the concept of the Shannon’s uniformity index (Shannon 1948), is introduced.

To illustrate the method, data concerning hemoglobin and transferrin, being blood polymorphic proteins, used as genetic markers for different production traits, e.g., prolificacy in sheep (Darcan and Güney 2001; Steppa 2005), are analyzed. Published linkage maps for the sheep genome present linkages of loci of the hemoglobin gene and FSHB hormone (Crawford et al. 1995; de Gortari et al. 1998). In turn, transferrin is a protein with a broad spectrum of functions in the organism, which may play a role in animal breeding due to its immunity functions. A certain role may also be attributed to the transport of iron ions, in which transferrin participates and which, in turn, may be further used in the synthesis of hemoglobin. A significant property of transferrin is its considerable heterogeneity, manifested in the capacity to identify in each population several alleles determining the occurrence of a large number of genotypes. A significant problem limiting the applicability of class I markers in forecasting prolificacy in sheep is connected with the difficulties in conducting a precise statistical analysis of such data with a discrete distribution.

The aim of this study was to show the utility of entropy analysis to characterize relationships between the prolificacy of dams and selected genetic attributes: the genotype of transferrin, the genotype of hemoglobin, and the type of birth, as well as the environmental attribute, i.e., year of birth. The entropy method may improve the exactitude of investigations concerning the influence of different factors on production trait.

## Materials and methods

Data were collected from four flocks of sheep, established and kept at the Swadzim Experimental Agricultural Station, Złotniki Farm, belonging to the Poznań University of Life Sciences (approved by Local Bioethical Committee, licence 39/2001 and 41/2002). Two of the analyzed flocks were prolific flocks: dairy sheep line 05 [flock 05] (81 % East Friesian dairy sheep, 19 % Polish Merino) and prolific sheep line 09 [flock 09] (44 % Polish Merino, 31 % East Friesian dairy sheep, 25 % Finnish sheep). The two other flocks are flocks of mutton type sheep: the White-headed mutton sheep [flock 06], considered to be a breed (50 % Texel, 18 % Ile de France, 9 % Berrichone du Cher, 11 % East Friesian dairy sheep, 6 % Polish Merino, 6 % Wielkopolska sheep), and a Dorset sheep line [flock 10] (31 % Dorset, 25 % Texel, 9 % Ile de France, 5 % Berrichone du Cher, 6 % East Friesian sheep, 21 % Polish Merino, 3 % Wielkopolska sheep).

In all the flocks, analyses were conducted on ewes born in the years 1990–2000. In both prolific flocks, the main selection traits at the selection of ewes for replacement were the type of birth of the ewe (single, twin, or triplet) and lifetime prolificacy of its dam. It was attempted to leave in the flocks the ewes from twin or triplet births, coming from dams, for which the lifetime prolificacy exceeded the flock average. In mutton flocks, the primary selection traits were growth rate and body weight of the ewe; additionally, similarly to in the prolific flocks, the parameters included in considerations were also the type of birth of the ewe and lifetime prolificacy of its dam. The first service for replacement ewes was performed in their first year of life at the age of approximately 10 months. All ewes were used for reproduction once a year.

Two class I genetic markers, i.e., hemoglobin and transferrin, were used in the analyses. Genotypes of hemoglobin (Hb) were identified using horizontal electrophoretic separation on starch gel (Smithies 1955), using buffers described by Gahne et al. (1960) and modified by Bojczuk (1984).

Genotypes of transferrin (Tf) were identified by horizontal electrophoretic separation in starch gel according to Smithies (1955), applying buffers described by Kristjansson (1963) and Gahne (1966) and modified by Bojczuk et al. (1980).

In each flock, the number of lambs born in the first, second, third, and fourth lambings was analyzed; for sheep barren in individual lambing dates (years), the number of lambs was taken to equal zero (Table 1).

**Table 1:** The number of ewes and mean numbers of lambs in the litter

Flock	Lambing 1		Lambing 2		Lambing 3		Lambing 4	
	*n*	Mean	*n*	Mean	*n*	Mean	*n*	Mean
05	252	1.194	229	1.463	166	1.783	121	1.678
06	269	1.052	242	1.112	193	1.363	147	1.354
09	231	1.446	211	1.687	160	1.788	108	1.843
10	151	1.033	131	1.229	102	1.441	75	1.373

Each ewe was characterized on the basis of the following attributes:

- Type of birth (single, twin, or triplet),
- Genotype of hemoglobin; three genotypes were found in each flock,
- Genotype of transferrin; 23 genotypes were found in flock 05, 21 genotypes in flock 09, 18 genotypes in flock 06, and 17 genotypes in flock 10,
- Calendar year of lambing as the environmental effect.

To establish the relationships within the data, entropy analysis was used in this study. The entropy *H*(*A*) of a discrete variable *A* measures the uncertainty connected with this variable:

$$ H(A)=-\sum\limits_a {p(a)} \ln p(a) $$

where *p*(*a*) denotes the probability of a given value of *A*. The value *H*(*A*) is an expected value of a discrete random variable, named information, taking values –ln *p*(*a*) with probabilities *p*(*a*). This variable has a property of taking great values for very rare events, but for the most certain events, it is close to zero. Conditional entropy *H*(*A*/*B*) quantifies the remaining uncertainty about *A* with the knowledge of *B*, i.e., $H\left( {A\left| B \right.} \right)=-\sum\limits_b {p(b)} \sum\limits_a {p\left( {a\left| b \right.} \right)} \ln p\left( {a\left| b \right.} \right) .$

For each pair of traits, the mutual information, namely:

$$ I\left( {A,B} \right)=H(A)+H(B)-H\left( {A,B} \right) $$

where $H\left( {A,B} \right)=H(A)+H\left( {B\left| A \right.} \right)=H(B)+H\left( {A\left| B \right.} \right)$ denotes the joint entropy, quantifies the interaction between attributes.

In the literature normed mutual information as a measure of dependency is given in two forms, namely:

$$ -U\left( {A,B} \right)=I(A,B)/[(H(A)+H\left( {B}) \right)/2] $$

known in the literature as Theil’s U (Mills 2011), and

$$  - J\left( {A,B} \right) = I(A,B)/H(A,B) $$

described by Jakulin (2005).

As mentioned previously, there are also different measures of variable interactions based on χ^2^ statistics. These are Pearson’s C, Cramer’s V, and Tschuprow’s T:

$$ \begin{array}{*{20}c} {C=\sqrt{{\frac{{{\chi^2}}}{{{\chi^2}+n}}}},} \hfill & {V=\sqrt{{\frac{{{\chi^2}}}{{n\min \left\{ {r-1,c-1} \right\}}}}},} \hfill & {T=\sqrt{{\frac{{{\chi^2}}}{{n\sqrt{{\left( {r-1} \right)\left( {c-1} \right)}}}}}}} \hfill \\ \end{array} $$

where *n* denotes the number of observations, and *r* and *c* are the numbers of rows and columns in the contingency tables, respectively.

Apart from entropy being the measure of uncertainty, literature sources provide the well-known Shannon diversity index, namely, *E*(*A*) = *H*(*A*)/ln(*s*_*A*_), where *s*_*A*_ is the number of categories of *A*. A generalization of this parameter on two variables may be a relative uniformity index, defined as:

$$ ED\left( {A,B} \right)=E(A)+E(B)-E\left( {A,B} \right) $$

where *E*(*A*,*B*) = *H*(*A*,*B*)/ln(*s*_*AB*_) and *s*_*AB*_ denotes the number of non-empty cells in a *s*_A_*s_*B*_ table. When *ED*(*A*,*B*) is close to one, both variables and their combinations are uniformly distributed in the population studied. When *ED*(*A*,*B*) is greater than one, it suggests a uniform distribution of at least one variable and a strong disuniformity of the combination of variables. The value less than zero indicates a disuniformity of *A* and *B* distributions, as well as their combinations, where disuniformity indicates significant differences in the frequencies of A and B combinations.

## Results and discussion

Table 2 presents estimators of previously described, entropy-based dependence measures between litter size and genotypes of hemoglobin and transferrin, the type, and year of birth of sheep. The analysis of these results indicates that environmental conditions (year) have the greatest effect on litter size in all the analyzed flocks and through all successive lambings. Flock 09, lambing 2, was the only exception in this respect, as the greatest amount of information on litter size was supplied by the genotype of transferrin. The considerable effect of the year on litter size was also shown in earlier studies conducted on the same flocks (Steppa 2005), when it was found that the effect of the year was manifested strongest in each flock in the first, second, and third lambings. Another trait providing the greatest amount of information in most cases was the genotype of transferrin, followed by the genotype of hemoglobin. It also needs to be stressed that these conclusions are identical for all the applied indexes I, J, and U. Steppa (2005), on the basis of factorial analysis of variance, stated that, in the examined flocks, there was a variation in litter size depending on the genotype of transferrin. The effect of this marker was strongest in the flock of prolific sheep line 09, in the first and third lambings, as well as the flock of the White-headed mutton sheep (06) in the second lambing. In the Dorset sheep line (10), in the first lambing, a considerable effect of this factor was also recorded. In the same analyses concerning the effect of the genotype of hemoglobin on litter size, also using factorial analysis of variance, the effect of the genotype of hemoglobin on litter size was observed only in the case of dairy sheep line 05 in the first and second lambings. Darcan and Güney (2001), who analyzed the effect of the genotype of hemoglobin and alleles of transferrin on litter size in Cukurova Assaf sheep, did not observe any effects of any of the above-mentioned factors.

**Table 2:** Measures of dependence for litter size and analyzed attributes

	Flock 05				Flock 09			
	I	J	U	ED	I	J	U	ED
Type of birth–lambing 1	0.038	0.024	0.046	0.626	0.018	0.010	0.020	0.669
Hb–lambing 1	0.051	0.028	0.054	0.744	0.013	0.007	0.013	0.778
Tf–lambing 1	0.075	0.026	0.051	0.623	0.069	0.021	0.041	0.663
Year–lambing 1	0.125	0.041	0.079	0.714	0.099	0.032	0.063	0.661
Type of birth–lambing 2	0.009	0.005	0.010	0.634	0.031	0.017	0.034	0.650
Hb–lambing 2	0.029	0.014	0.028	0.746	0.018	0.009	0.018	0.764
Tf–lambing 2	0.078	0.026	0.050	0.706	0.115	0.034	0.066	0.649
Year–lambing 2	0.122	0.037	0.072	0.710	0.094	0.030	0.059	0.683
Type of birth–lambing 3	0.012	0.008	0.015	0.552	0.029	0.018	0.034	0.699
Hb–lambing 3	0.030	0.017	0.033	0.720	0.036	0.020	0.040	0.731
Tf–lambing 3	0.099	0.035	0.068	0.666	0.126	0.039	0.074	0.660
Year–lambing 3	0.110	0.037	0.071	0.710	0.136	0.050	0.094	0.679
Type of birth–lambing 4	0.006	0.004	0.008	0.641	0.041	0.022	0.043	0.679
Hb–lambing 4	0.031	0.017	0.033	0.739	0.047	0.023	0.045	0.813
Tf–lambing 4	0.093	0.032	0.062	0.717	0.173	0.055	0.104	0.677
Year–lambing 4	0.107	0.036	0.069	0.767	0.177	0.064	0.121	0.692
	Flock 06				Flock 10			
Type of birth–lambing 1	0.005	0.004	0.005	0.901	0.001	0.001	0.001	0.891
Hb–lambing 1	0.006	0.004	0.005	0.702	0.006	0.004	0.008	0.601
Tf–lambing 1	0.032	0.013	0.010	0.773	0.095	0.030	0.059	0.776
Year–lambing 1	0.117	0.040	0.026	0.794	0.141	0.049	0.094	0.731
Type of birth–lambing 2	0.007	0.004	0.005	0.935	0.023	0.016	0.031	0.818
Hb–lambing 2	0.011	0.007	0.008	0.712	0.011	0.007	0.015	0.550
Tf–lambing 2	0.037	0.014	0.011	0.819	0.097	0.031	0.061	0.642
Year–lambing 2	0.115	0.038	0.024	0.866	0.133	0.046	0.089	0.607
Type of birth–lambing 3	0.007	0.004	0.005	0.835	0.019	0.012	0.023	0.830
Hb–lambing 3	0.013	0.008	0.010	0.615	0.018	0.011	0.022	0.590
Tf–lambing 3	0.054	0.021	0.015	0.683	0.101	0.033	0.064	0.702
Year–lambing 3	0.070	0.023	0.015	0.702	0.157	0.054	0.102	0.716
Type of birth–lambing 4	0.006	0.004	0.005	0.915	0.015	0.009	0.019	0.830
Hb–lambing 4	0.030	0.019	0.024	0.749	0.012	0.007	0.015	0.590
Tf–lambing 4	0.034	0.014	0.011	0.793	0.148	0.052	0.099	0.716
Year–lambing 4	0.076	0.027	0.018	0.800	0.178	0.064	0.120	0.722

*I* mutual information, *J* Jakulin’s measure, *U* Theil’s U, *ED* relative uniformity index, *Hb* hemoglobin, *Tf* transferrin

The estimated relative uniformity indexes indicate a similarity between flocks 05 and 09, in which this index assumes the highest value, approaching one for the genotype of hemoglobin in combination with litter size. For flocks of mutton sheep 06 and 10, the highest value of *ED*(*A*,*B*) was obtained for type of birth and litter size. This indicates a uniform distribution of frequencies over categories. It may be assumed that the similarity between the prolific flocks shown on the uniformity index *ED*(*A*,*B*) is the result of the genetic similarity of sheep in both flocks—in breeding work on the generation of both populations, common breed components were used—the East Friesian sheep and the Polish Merino. The mutton sheep are characterized by genetic discreteness in comparison to the prolific flocks. In the genotype of both mutton flocks, genes of imported European mutton breeds predominate. It also needs to be stressed that, in the flocks of the prolific and mutton sheep, other selection criteria were applied in selection for flock replacement. The results prepared for both mutton flocks also indicate that the type of birth of the ewe, being in mutton sheep an additional selection criterion, had the greatest effect on litter size.

The analysis of results concerning the dependence between three attributes, i.e., the type of birth, the genotype of hemoglobin, and the genotype of transferrin given in Table 3, shows a similarity between flocks 05, 06, and 10, in which the dependence between the genotypes of transferrin and hemoglobin predominates. An exception in this respect was provided by flock 09, where the greatest value of the index was recorded for the type of birth and the genotype of transferrin. However, estimated dependencies between the type of birth and transferrin, as well as hemoglobin and transferrin, in all the analyzed flocks are similar. Markedly lower estimators were obtained for the dependencies between the type of birth and hemoglobin. It needs to be observed that all the proposed indexes still provide the same response.

**Table 3:** Measures of variable interactions

	Flock 05				Flock 09			
	I	J	U	ED	I	J	U	ED
Type of birth–Tf	0.071	0.023	0.023	0.714	0.128	0.037	0.035	0.844
Type of birth–Hb	0.014	0.008	0.008	0.788	0.013	0.007	0.007	0.858
Hb–Tf	0.093	0.029	0.028	0.875	0.094	0.026	0.025	0.939
	Flock 06				Flock 10			
Type of birth–Tf	0.041	0.015	0.015	0.952	0.036	0.011	0.011	0.994
Type of birth–Hb	0.003	0.002	0.002	0.869	0.019	0.013	0.013	0.880
Hb–Tf	0.049	0.018	0.018	0.680	0.087	0.027	0.026	0.758

*I* mutual information, *J* Jakulin’s measure, *U* Theil’s U, *ED* relative uniformity index, *Hb* hemoglobin, *Tf* transferrin

In the analysis of the uniformity index, we may see, again, a distinct difference between the prolific and mutton flocks. In the first group, the dependence between the genotype of transferrin and the genotype of hemoglobin predominates, followed by the genotype of transferrin–type of birth and type of birth–genotype of hemoglobin dependencies. In the flocks of mutton sheep, the ordering is opposite, indicating the highest uniformity of the distribution for the type of birth and genotype of transferrin combination. Thus, the ED coefficient indicates a separateness of the prolific and mutton flocks. The high consistency of estimations for the indexes described in this study is also shown by values of linear correlation coefficients close to or exceeding 0.95 (Table 4). Similarly, high correlations were observed in the group of indexes based on traditional methods. Between the groups of methods, these indexes are slightly lower, but not lower than 0.85.

**Table 4:** Correlation coefficients for the discussed measures of dependencies

Measure	I	J	U	C	V
Jakulin’s J	**0.968**				
Theil’s U	**0.942**	**0.977**			
Pearson’s C	0.934	0.898	0.851		
Cramer’s V	0.891	0.868	0.823	**0.967**	
Tschuprow’s T	0.850	0.870	0.824	**0.942**	**0.968**

## Conclusions

The consistency between the actual status (the genetic share of breed components in individual flocks and selection methods) and the results of analyses confirms the appropriateness of the adopted methods of statistical analysis. The index of relative uniformity introduced in this study thus proved to be an adequate tool for the determination of similarity, as well as a lack of similarity in the examined flocks. Moreover, the application of mutual information in the determination of values of the dependence measures in the analyzed experiment was particularly justified due to the high number of transferrin cells with zero values.
